# Mortality predictors of patients suffering of acute pancreatitis and development of intraabdominal hypertension

**DOI:** 10.3906/sag-1809-15

**Published:** 2019-04-18

**Authors:** Maja STOJANOVIC, Petar SVORCAN, Aleksandar KARAMARKOVIC, Nebojsa LADJEVIC, Radmilo JANKOVIC, Predrag STEVANOVIC

**Affiliations:** 1 Department of Anesthesiology and Intensive Care, “Zvezdara” University Medical Center, Belgrade Serbia; 2 Faculty of Medicine, University of Belgrade, Belgrade Serbia; 3 Department of Gastroenterology and Hepatology, “Zvezdara” University Medical Center, Belgrade Serbia; 4 Department of Surgery, “Zvezdara” University Medical Center, Belgrade Serbia; 5 Department of Anesthesiology and Intensive Care, Clinical Center of Serbia, Belgrade Serbia; 6 Department of Anesthesiology and Intensive Care, Faculty of Medicine, University of Niš, Niš Serbia; 7 Department of Anesthesiology and Intensive Care, Clinical Center of “Dr Dragiša Mišović”, Belgrade Serbia

**Keywords:** Acute pancreatitis, intraabdominal pressure, intraabdominal hypertension, shock, critical care, mortality

## Abstract

**Background/aim:**

Intraabdominal hypertension (IAH) occurs frequently in patients with acute pancreatitis and adds to their morbidity and mortality. The main aim of the study was to identify the determination of the predictive factors connected to IAH that influence the evolution of acute pancreatitis.

**Materials and methods:**

The prospective cohort study was conducted on 100 patients who had acute pancreatitis. According to obtained intraabdominal pressure (IAP) values, the patients were divided into two groups: one group (n = 40) with normal IAP values and the other (IAH group, n = 60) with increased IAP values. Deceased patients were specially analyzed within the IAH group in order to determine mortality predictors.

**Results:**

Statistical significance of IAP (P = 0.048), lactates (P = 0.048), peak pressure (P = 0.043), abdominal perfusion pressure (P = 0.05), and mean arterial pressure (P = 0.041) was greater for deceased than for surviving patients in the IAH group. High mortality appears for patients younger than 65 years old, with lactate level higher than 3.22 mmol/L and filtration gradient (GF) lower than 67 mmHg.

**Conclusion:**

Age, lactates, GF, and APACHE II score are determined as mortality predictors for patients suffering from acute pancreatitis who developed IAH. The mortality rate is higher when the level of GF is decreasing and the level of lactate increasing.

## 1. Introduction

A number of studies show that intraabdominal hypertension (IAH) and abdominal compartment syndrome (ACS) are significant indicators of the morbidity and mortality of patients with acute pancreatitis (1–3). Massive fluid replacement in the early stage of acute pancreatitis combined with inflammatory processes in the retroperitoneum results in visceral edema leading to early organ damage and gradual development of IAH. An increase in intraabdominal pressure (IAP) above 12 mmHg leads to perfusion disorder of the intestinal organs, which, along with the existing microcirculatory disorder of the pancreas, leads to reduced oxygen delivery, development of necrotic formations in the pancreas, cytokine release, sepsis development, and deterioration of the patient’s condition (4,5). Presence of comorbidity (chronic renal insufficiency, lung disease, cardiomyopathy) plays a significant role in the deteriorating effects of high IAP values and lowering threshold levels of IAH, which leads to clinical manifestations of ACS. This syndrome has a very high mortality rate, especially due to development of sepsis and multiorgan failure (6,7).

The maximum IAP; the Acute Physiological, Age, and Chronic Health Evaluation II (APACHE II) score; the maximum sequential organ failure assessment score; age; maximum lactate and creatinine; and base deficiency were previously found to be significantly increased in deceased patients with acute pancreatitis (8,9). The aim of this study is to determine the predictive factors for development of IAH, which influences the evolution of acute pancreatitis. 

## 2. Materials and methods

The current research was designed as a prospective cohort study conducted from January 2016 to December 2017 in the Intensive Care Unit (ICU) of “Zvezdara” University Medical Center in Belgrade. The study was approved by the local medical ethics committee (Clinical Center Zvezdara IRB 1 - Medical, No. IRB00003818, approved by Federalwide Assurance - FWA00006109). All the participants or their legal guardians were provided with information regarding the study protocol and provided signed consent prior to the study.

The risk group included 100 patients with acute pancreatitis developed for the first time (there were no recurrent pancreatitis cases). The etiology of acute pancreatitis was biliary (sludge, microlithiasis, or gallstones) in 48 patients, alcoholic in 24, hypertriglyceridemic in 20, and of idiopathic origin in 8. This study does not cover previously operated patients or patients with trauma, burns, liver dysfunction (cirrhosis with ascites), acidosis (pH > 7.2), hypothermia (temperature < 33 °C), intraabdominal or retroperitoneal tumors, gastroparesis, gastric distension, ileus, chronic kidney failure, or peritoneal dialysis. The diagnostic criterion for acute pancreatitis is defined as the presence of two out of three conditions: typical abdominal pain, a threefold increase of serum amylase or lipase, and verification of findings for unclear cases of acute pancreatitis on CT examination (10). Based on the World Society of the Abdominal Compartment Syndrome (WSACS) recommendations, IAH is defined as the constant presence or repeated pathological increase of IAP at 12 mmHg or higher (4).

Patients (n = 100) were divided into two groups according to the measured values of IAP and monitored until they left the ICU. In one group were the patients with normal values of IAP (n = 40) and in the other, the IAH group, were the ones with elevated values of the IAP (n = 60). In each of these groups basic vital functions parameters were separately analyzed and compared concerning IAP values. Deceased patients within the group with IAH were separately analyzed, and in order to assess the probability of mortality, APACHE II score was determined, as well as the values ​​of abdominal perfusion pressure (APP) and filtration gradient (GF). 

Measured IAP values were compared with age, sex, body mass index (BMI), number of days of treatment, mean arterial pressure (MAP), central venous pressure (CVP), respiratory rate per minute, level of lactate, APACHE II score, APP, and GF. All variables were measured upon admission to the ICU, and then each 12 h until the patient was transferred to another department. Average values were determined for all variables, as well as average values with respect to number of days of treatment in the ICU. 

### 2.1. Measuring of the intraabdominal pressure

The IAP was measured according to WSACS recommendations, in the way described in a previous study (1). Patients had a urinary catheter placed in the bladder, whose other end was connected by a three-way stopcock to the urine bag and the hose of the infusion set, attached to a measuring ruler calibrated in centimeters. During the measurement the bag was taken off and 25 mL of the sterile saline solution was injected into an empty bladder through the hose of the infusion set. The measurement was performed at the end of expiration, 30–60 s after the injection of the solution, with the patient lying down still, horizontally on his or her back. Zero point was on the level of the medium axillary line on the iliac crest. The IAP was recorded every 12 h and the obtained values were expressed in mmHg using a correction factor of 1 mmHg = 1.36 cmH2O.

### 2.2. Statistical analyses

Data analysis was conducted using SPSS 23 (IBM Corp., Armonk, NY, USA) and Statistica version 13 for Windows (StatSoft, Tulsa, OK, USA). Categorical variables are given in numbers and percentages, while discontinuous and continual characteristics (series) are presented as medium values and standard deviations. Student’s t-test was used to compare differences between two groups of parametric data for a single characteristic. The chi-square test was used for the distinction analysis between two groups of nonhomogeneous or nonparametric data based on one characteristic. The significance of the difference of the individual characteristic values (shown as middle values) was estimated between the two studied groups. 

Determination of the statistical significance of IAP for the change of variables within a particular IAP group is shown by analyses of the variance in the regression model. The regression analyses that were used were simple linear and curvilinear, simple multilinear regression, and stepwise regression. For the purpose of interpreting potential combinations of susceptibility and specificity for possible predictors, the receiver operating characteristic (ROC) curve was used and the area under the curve (AUC) was displayed for mean values. The AUC ranges from 0 to 1 and represents the degree of modeling ability in the separation of entities that experienced the observed event in relation to those that did not. In all applied statistical methods, the level of significance was P ≤ 0.05. 

## 3. Results

### 3.1. Descriptive analysis of the examined patients

The study covers 100 patients, of which 40 had normal IAP values and 60 had IAH. Descriptive analysis of the examined patients is presented in the Table.

**Table:** Descriptive analysis of studied patients.

Study variables	Patients with normalIAP (n = 40)	Patients withIAH (n = 60)	P-value
IAP, mmHg	8.3 ± 2.3	16.4 ± 4.2	0.004
Age, years	67.5 ± 14.8	65.4 ± 12.3	0.623
Sex, male : female	21 : 19	38 : 22	0.036
Number of deceased, n (%)	3 (7.5%)	28 (46.7%)	0.023
BMI, kg/m2	23.99 ± 3.4	25.9 ± 2.5	0.001
Number of days of treatment	5.8 ± 6.2	8.4 ± 8.6	0.195
APACHE II score	27.86 ± 7.4	29.2 ± 6.65	0.491
MAP, mmHg	88.35 ± 15.4	94.15 ± 16.75	0.001
CVP, cm H2O	9.24 ± 3.48	12.77 ± 4.79	0.003
Respiratory rate/min	18.48 ± 4.6	23.3 ± 7.4	0.002
Lactate, mmol/L	1.2 ± 0 .57	3.22 ± 2.07	0.001
APP, mmHg	80.87 ± 16.48	79.8 ± 16.05	0.002
GF, mmHg	71.18 ± 17.2	67.67 ± 18.49	0.006

IAP: Intraabdominal pressure; IAH: intraabdominal hypertension; BMI: body mass index; APACHE II: Acute Physiology, Age, and Chronic Health Evaluation II; MAP: mean arterial pressure; CVP: central venous pressure; APP: abdominal perfusion pressure; GF: filtration gradient.

### 3.2. Differences between variables for deceased and surviving patients within the IAH group

Within the IAH group there were 28 deceased patients and 32 surviving ones. Using the nonparametric chi-square test, statistically significant differences were found for the following variables: IAP (P = 0.048), lactates (P = 0.048), peak pressure (P = 0.043), APP (P = 0.05), and MAP (P = 0.041). 

Using stepwise regression analysis, three variables that were crucial in monitoring mortality rates were identified: GF, lactates, and age. In our case, the mortality rate for patients with high IAP could be calculated using the following formula:

 Y = 28.430 - 0.158 × GF + 0.975 × lactates - 0.065 × age,

where GF is the filtration gradient. 

**Figure 1:**
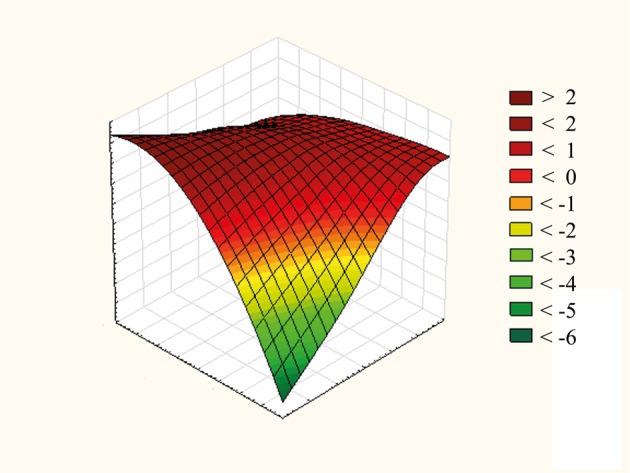
Effect of APACHE II score and MAP on disease outcome in patients with IAH. APACHE II: Acute Physiology, Age, and Chronic Health Evaluation II; MAP: mean arterial pressure; IAH: intraabdominal hypertension.

**Figure 2:**
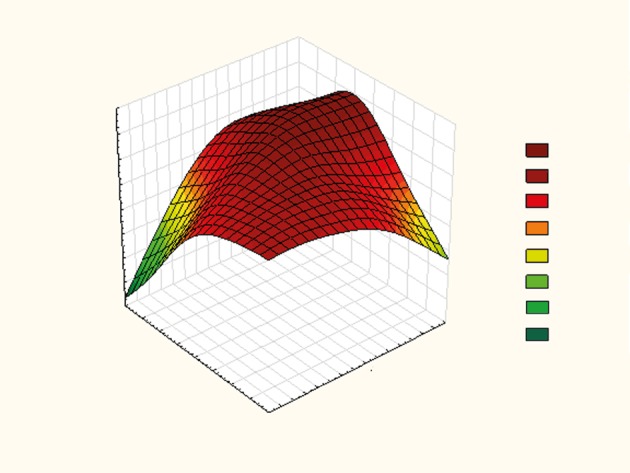
Effect of APACHE II score and CVP on disease outcome in patients with IAH. APACHE II: Acute Physiology, Age, and Chronic Health Evaluation II; CVP: central venous pressure; IAH: intraabdominal hypertension.

**Figure 3:**
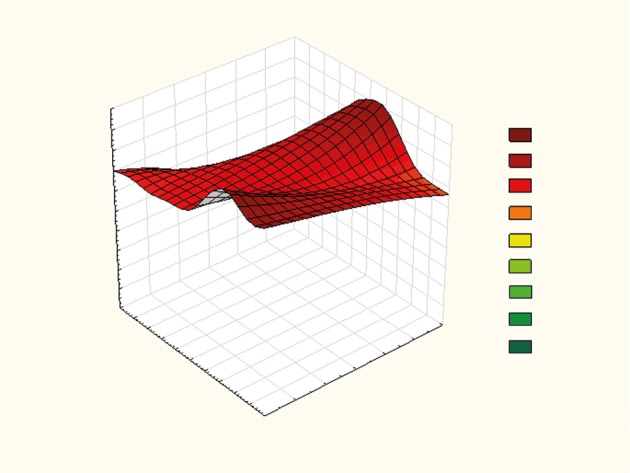
The effect of lactate and GF on disease outcome in patients with IAH. GF: Filtration gradient; IAH: intraabdominal hypertension.

Simple multilinear regression shows that the disease outcome is influenced by APACHE II score and MAP (Figure 1), by APACHE II score and CVP (Figure 2), and by lactates and GF among patients with IAH (Figure 3). We obtained formulas for calculating the mortality rate of our patients with high IAP as a function of the examined variables as follows:

Y = 2.108 – 0.017 × MAP + 0.034 × APACHE II,

Y = –0.267 + 0.047 × APACHE II + 0.029 × CVP,

Y = 3.004 – 0.051 × lactate – 0.025 × GF,

where MAP is mean arterial pressure; APACHE II is the Acute Physiology, Age, and Chronic Health Evaluation II score; CVP is central venous pressure; and GF is the filtration gradient. 

### 3.3. Determination of mortality predictors among patients with IAH

In order to determine mortality predictors, ROC curves and AUCs for middle values are shown.

**Figure 4:**
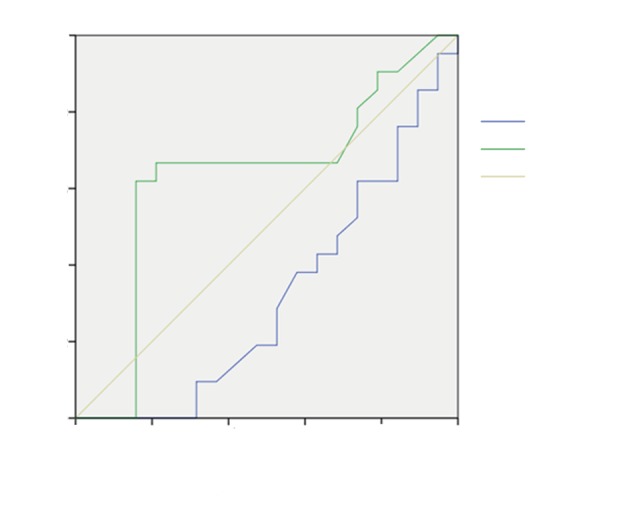
Receiver operating characteristic (ROC) curve prediction of age and GF in patients with IAH. GF: Filtration gradient; IAH: intraabdominal hypertension.

Figure 4 shows the effect of age and GF for patients with IAH, along with AUCs for mean values of the age of 0.317 years (95% confidence interval (CI): 0.149–0.486) and GF of 0.630 mmHg (95% CI: 0.444–0.816). GF was singled out as a predictor of mortality as it increases together with the mortality rate of patients with elevated IAP.

**Figure 5:**
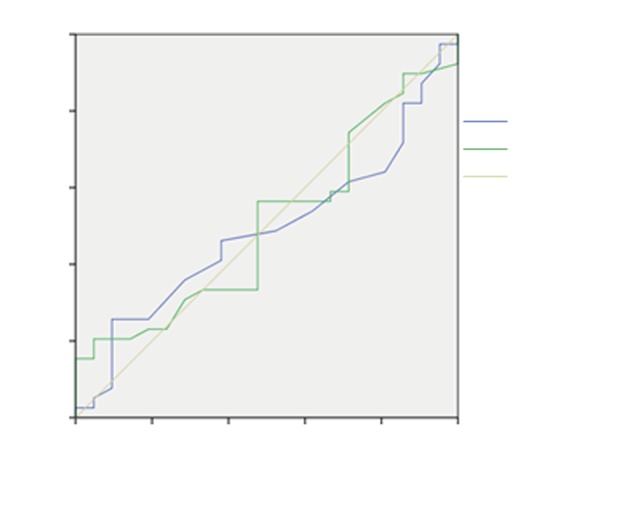
Receiver operating characteristic (ROC) curve prediction for APACHE II score and lactate in patients with IAH. APACHE II: Acute Physiology, Age, and Chronic Health Evaluation II; IAH: intraabdominal hypertension.

The ROC curve and AUC is shown in Figure 5 for prediction of mortality of patients with IAH for APACHE II score and lactate variables. It shows a strong correlation of APACHE II score and lactate values to IAP, as increase of these values follows the increase of mortality rate. The AUC for mean values of the APACHE II score and lactate are 0.513 (95% CI: 0.364–0.662) and 0.529 mmol/L (95% CI: 0.379–0.680), respectively. APACHE II score and lactate were found to be predictors of mortality in patients with high levels of IAP. 

## 4. Discussion

IAH has 50% prevalence among critically diseased patients with acute pancreatitis in the ICU (11). It is one of the risk factors that can lead to death and increased mortality rate (9,12). Large numbers of patients suffering from different diseases (acute pancreatitis, acidosis, major burns, major trauma, distended abdomen, massive incisional hernia repair, sepsis, bacteremia, intraabdominal infection) can develop IAH (1). The severest consequences that prolong ICU treatment, raise treatment prices, and interfere with normal functioning of patients are ACS, multiorgan failure, and death. Timely identification of patients suspected of developing IAH can prevent severe consequences of this syndrome, through monitoring of the IAP with simple methods and protocol treatment of developed IAH. 

In our study group, 60 of 100 patients had developed IAH. Compared to patients with normal IAP, patients with IAH had much higher average IAP values and were tachypneic and younger with male predominance. They also had higher lactate values, BMI, MAP, and CVP and lower APP and GF values. There were no significant differences between average values of APACHE II scores and the number of treatment days. 

The study conducted by Muturi et al. (13) had a similar representation between men and women as our study, with male predominance and higher IAP values, but a younger age (37 years). Another study that included patients under the age of 30 who were not previously ill showed that sex had no influence on IAP values (14). It was previously shown that increased BMI leads to the increase of IAP (15–18) and an increase of 1 kg/m2 in BMI leads to the increase of 0.07 mmHg of IAP (15). The increase of BMI is correlated to the increase of IAP, but it was also shown that it does not depend on sex or race (15). 

Total mortality rate of both of our groups was 31% (31 out of 100 patients): among patients with normal IAP values mortality was 7.5% (3 out of 40), while in the IAH group it was 46.7% (28 out of 60). Comparison of disease outcomes showed that there was a higher mortality rate of patients with IAH with respect to patients with normal IAP (P = 0.023). The increase of IAP led to changes in the functioning of other organ systems, regarding multiorgan syndrome, and consequently led to a higher mortality rate.

As most of our patients had IAH and those patients had a higher mortality rate, further determination of predictive factors was conducted within this group. The goal was to identify changes in a patient’s condition as early as possible in order to provide adequate treatment response. Deceased patients had much higher average IAP values, increased lactate values, inadequate respiration mechanism, and poorer gas exchange and consequently had a greater need for mechanical ventilation. These patients additionally suffered an increase in IAP due to positive pressure ventilation and thus had higher values of peak pressure. Inadequate respiratory mechanism, a greater need for mechanical ventilation, and higher peak pressure values of those patients were also demonstrated in other studies (18–20). Also, for patients who died, the increase of IAP was followed by a decrease of APP due to inadequate organic perfusion that led to even greater lactate values. Lower values of MAP that we found were most likely a consequence of the increase of IAP, which induces increased pressure in the chest and the compression of the organs of that cavity.

It is known that kidney function disorder is almost inevitable in patients with IAH (21), but some recent studies indicated that kidney injury may already occur at low values of IAP and not just in developed ACS cases (22,23). Values of IAP from 10 to 15 mmHg directly act on the kidneys, provoking compartment syndrome in the kidney itself (23). With further increase the IAP acts on kidney veins (leading to decrease in kidney outflow), on the abdominal aorta and kidney arteries (leading to increase of kidney vascular resistance), and on redistribution of the kidney cortex towards the medulla (leading to lowered glomerular filtration, which is the best indicator of renal impairment) (21,22). Our study shows that one of the predictors of mortality is precisely the parameters of kidney function.

Modern statistical methods were applied in order to determine predictive factors of mortality. Three factors were singled out, namely GF, lactates, and age of patients. Increased IAP results in decreased organ perfusion, either due to compressive action on the abdominal organs or induced pressure in another compartment and additional circulation disorders in these organs. Reducing the perfusion of splanchnic circulation due to further increase in IAP leads to reduction of GF and diuresis per hour and, with developing ACS, anuria. Inadequate organ perfusion and excretion from the organism leads to accumulation of toxic and decomposed products and high level of lactates. Also, all of these changes could appear even in younger patients, as in our study the patients with IAH were younger than the ones with normal IAP. If diuresis per hour decreases and lactates values increase, especially for younger patients, one should suspect IAH development and apply treatment procedures immediately in order to prevent death. 

Combinations of predictive factors that lead to the increase of mortality rate were also analyzed. For the purpose of obtaining valid statistical data, predictive factors were determined for the IAH group because of the high number of deceased patients. Increasing of APACHE II scores up to a maximum of 40 and MAP up to a maximum of 120 mmHg leads to the increase of mortality rate. Their mutual interaction, expressed as a simple function (Y = 2.108 – 0.017 × MAP + 0.034 × APACHE II), enables us to predict the number of deaths. Increasing CVP to 28 and APACHE II score to 40, the mortality rate will be the highest. Following these variables on a daily basis, one can calculate the probability of death, making it possible to take urgent measures to reduce the IAP.

Lactates and GF, alone or combined, lead to a very high mortality rate, especially if combined, because they show a very high correlation. We can safely identify them as the factors that can help determine the degree of mortality: reducing the GF value and increasing the lactate value increases the mortality rate.

In order to find mortality predictors and once more confirm them, analysis of ROC curves was employed (the area under the curves determines the influence of the investigated variables on the degree of mortality). It was found that age has an impact on the change in the value of IAP. Raised IAP values lower GF values, too. Among patients with IAH, age and GF in combination are determined as mortality predictors. 

Likewise, APACHE II score was separated as a mortality predictor, because it greatly affects the change of IAP values, especially among patients with IAH. Patients with IAH already have additionally damaged organ systems, besides the dominant effect of high IAP values. The combination of high APACHE II scores and high IAP values increases the mortality rate. The variable of lactates was also separated as one of the mortality predictors. It was found that the increase of IAP values leads to the increase of lactates and the result of their combined influence is the increase of the mortality rate.

In conclusion, we determined variables that predict high mortality of patients with IAH: age of 65 years and less, lactate level higher than 3.22 mmol/L, GF rate lower than 67 mmHg, and APACHE II score higher than 29. The mortality rate increases the most when the level of GF is decreasing and the level of lactate increasing. IAH is commonly present in the ICU and has the great influence on the survival rate. Therefore, the obligation of all doctors in the ICU should be monitoring IAP among patients suspected to develop IAH or ACS. There are several reasons for that: IAH is very frequently present in critically diseased patients; IAH is very important because it has high morbidity and mortality rates; IAH can be detected in the early phase; and IAH and ACS can be treated. Therefore, the aim is to recognize such patients, not only for the sake of lowering IAP, but also in order to improve organ function and decrease the mortality rate.
